# Psychophysiological Factors Moderate Amygdala–Prefrontal Connectivity Linked to Perceived Peer Victimization and Depressive Symptoms in Preadolescent Migrant Children

**DOI:** 10.1155/2024/5596651

**Published:** 2024-10-10

**Authors:** Xinyu Gong, Ting Tian, Jiahua Xu, Shaozheng Qin, Danhua Lin

**Affiliations:** ^1^Institute of Developmental Psychology, Beijing Normal University, Beijing, China; ^2^State Key Laboratory of Cognitive Neuroscience and Learning and IDG/McGovern Institute for Brain Research, Beijing Normal University, Beijing, China; ^3^Beijing Key Laboratory of Brain Imaging and Connectomics, Beijing Normal University, Beijing, China; ^4^Psychiatry Research Center, Beijing Huilongguan Hospital, Peking University Huilongguan Clinical Medical School, Beijing, China

**Keywords:** amygdala functional connectivity, cortisol, depressive symptoms, perceived peer victimization, self-esteem

## Abstract

**Background:** As a sense of an intense stressor, perceived peer victimization can cause adverse effects on mental health, like depressive symptoms. Yet, little is known about the neurobiological mechanisms underlying how perceived peer victimization causes and maintains depressive symptoms in preadolescence.

**Methods:** Here we investigate the effects of peer victimization on amygdala subregional functional connectivity in 101 preadolescent migrant children, and their relations to depressive symptoms and potential protective factors of self-esteem and daily cortisol. Further control analyses were conducted to verify whether there are any specific effects in migrant children compared to 54 age-matched preadolescent children from nonmigrant background.

**Results:** Children with higher perceived peer victimization exhibited greater intrinsic functional connectivity of the amygdala with the middle frontal gyrus extending into the superior frontal gyrus. Perceived peer victimization could account for an indirect association between amygdala hyperconnectivity and depressive symptoms. Moderated mediation analyses revealed that basolateral amygdala connectivity with the superior frontal gyrus acted as a neural marker linking peer victimization and greater risk for depressive symptoms only in preadolescent children with low self-esteem or low daily cortisol.

**Conclusions:** These findings suggest that considering neurobiological vulnerability and psychophysiological factors may gain a nuanced understanding of the adverse effects of perceived peer victimization on depressive symptoms, a risk for internalizing pathology. This study could inform personalized intervention strategies to prevent or ameliorate depressive symptoms in this disadvantaged population.

## 1. Introduction

As a sense of an intense stressor, perceived peer victimization, which reflects the perceived aggression of peers, has far-reaching effects on individuals' psychopathology and socioemotional functioning [1]. The prevalence rate of students' perceived peer victimization worldwide is up to 30% [2]. It is noteworthy that rural-to-urban migrant children increase their sensitivity to peer relationships due to disadvantaged social environments, like abuse and poverty, thus more vulnerable to experiencing peer victimization [3]. However, little is known about the neurobiological mechanisms underlying how perceived peer victimization causes among migrant children.

Consistent with the stress generation model [4], individuals with higher neural susceptibility to stress can have a higher probability to perceive stress. Hyperconnectivity of the amygdala with the prefrontal cortex (PFC) regions at the resting state has been revealed to be vulnerable to stress [5], suggesting a potential link between the amygdala–prefrontal connectivity and perceived stress pertaining to peer victimization. Indeed, one neuroimaging study has shown that higher amygdala-PFC connectivity was related to poorer emotion regulation and elevated perception of peer victimization in adolescent girls [6]. Other studies have indicated that heighted mPFC-amygdala connectivity is associated with more difficulties in emotion regulation, which may increase the susceptibility to peer victimization [7].

Preadolescence refers to the developmental stage that precedes adolescence, broadly encompassing children in the age range of approximately 9–13 years old [8]. This phase marks the beginning of the transition from childhood to adolescence, where preadolescent children are poised at the threshold of puberty and experiencing a heightened sensitivity to peer relationships and stress [9, 10]. Moreover, both childhood and adolescence are sensitive periods for the amygdala and prefrontal development [9], the amygdala–prefrontal connectivity also goes through critical development during childhood and adolescence, with being positive at first in childhood and switching to relatively negative during the transition to adolescence [11]. For instance, there are studies showing that among participants under 10 years old, the amygdala-medial PFC functional connectivity is significantly positive, whereas for those aged 10 and above, the functional connectivity turns significantly negative [12]. Given the age characteristics, the first purpose of the study is to examine the potential association between the resting-state amygdala–prefrontal circuitry and perceived peer victimization among preadolescent migrant children.

Furthermore, the centromedial amygdala (CMA) and basolateral amygdala (BLA), as two major subregions of the amygdala, have been demonstrated to play a distinct role in emotion processing [13]. Specifically, BLA is critical in the input processing of emotional information, while CMA mainly generates emotional responses [14]. While deficits in emotional processing may contribute to the occurrence of perceived victimization by peers [15], implying the distinct role of these two amygdala subregions in modulating the information perception of peer victimization. Therefore, amygdala nuclei seed-based functional connectivity has been employed to facilitate our deep understanding of the resting-state brain activity related to perceiving peer victimization among preadolescent migrant children.

One fundamental question in the neuroscience of socioemotional stress is to understand how the emotional circuitry along with subjectively perceived social pressure may cause and maintain psychopathology in susceptible individuals [16]. Given that the amygdala–prefrontal circuitry may sensitize perception and regulation of emotional and stressful events, and perceived peer victimization is supported by abundant evidence to be associated with a higher risk for depressive symptoms during childhood and adolescence [17, 18]. It is thus necessary to clarify potential associations and mediating paths among emotion circuitry, perceived peer victimization, and depressive symptoms. Recently, increasing evidence from neuroimaging studies demonstrates that subjectively perceived stress may play an important role in the link between amygdala–prefrontal brain circuitry and internalizing problems [19]. It is conceivable to speculate the potential effect of perceived peer victimization on the relationship between the amygdala–prefrontal circuitry and depressive symptoms. Therefore, the second purpose of our study is to explore whether perceived peer victimization mediates the relationship between amygdala–prefrontal connectivity and depressive symptoms in preadolescent migrant children. Considering that many empirical studies have found that adversity could affect the brain activity and lead to internalizing problems, our study would also do the competition model whether amygdala–prefrontal hyperconnectivity mediates the association between perceived peer victimization and depressive symptoms.

Last but not least, identifying joint mechanisms of protective factors and stressor-induced changes in functional connectivity in the perception of peer victimization is expected to provide entry points for individualized treatment and possibly prevention. Self-esteem, as an important psychological protective factor, is often described as a subjective evaluation of an individual's worth as a person [20]. According to the cognitive diathesis-stress model of depressive symptoms [21], cognitive diathesis plays an important role in regulating depressive symptoms. Self-esteem, as one kind of positive cognitive diathesis, is vital to protecting individuals from perceiving stressful events [22]. Empirical research has supported that higher self-esteem may be a buffer to the toxic effects of neural vulnerability to perceived stress [23]. Besides, the stress hormone cortisol, as an end-product of the hypothalamic–pituitary–adrenal axis (HPA axis) in response to stressful events, is essential for the body to prepare for the daily environmental demands [24]. Notably, the modulation of cortisol levels is involved in the amygdala–prefrontal coupling to achieve adaptive brain responses to stress and help maintain homeostasis [25]. The morning daily cortisol contains important diurnal features of the HPA-axis activity, with moderate higher morning cortisol level beneficial to provide daily energy support and link with lower levels of fatigue and physical symptoms later for that day [26] but chronic excessive morning cortisol has a close link with a variety of mental outcomes, such as depressive symptoms and subjectively perceived stress [27]. Indeed, participants with less depressive symptoms consistently have higher morning cortisol levels compared to those with more depressive symptoms [28], which may mean higher morning cortisol levels help provide more energy for the environmental demands and regulate mood states, suggesting a potential buffering role of higher morning cortisol among the relationships of the emotion-related amygdala–prefrontal connectivity, peer victimization perceiving and depressive symptoms [25]. Nonetheless, whether these two psychophysiological factors (i.e., high self-esteem and higher cortisol levels) could buffer the association between the amygdala–prefrontal functional organization and perceived victimization in preadolescent migrant children has not been clarified yet.

To address the above three issues, we implemented resting-state functional magnetic resonance imaging (fMRI) along with psychological and endocrinal assessments in 101 migrant children aged from 10 to 14 years old. Resting-state functional connectivity (RSFC) of fMRI data is recognized as a promising approach for identifying the neurobiological signatures linked to emotional and mental health problems [29]. Multiple regression analyses were performed to uncover a neurofunctional basis (mainly focused on amygdala functional connectivity) of perceived peer victimization. Moreover, mediation analyses as well as moderated mediation analyses were performed to explore the buffering role of self-esteem and morning daily cortisol on the adverse effects of amygdala–prefrontal connectivity on perceived peer victimization and depressive symptoms.

## 2. Methods

### 2.1. Participants

A total of 124 urban migrant Chinese children, aged from 9 to 14 (60 boys and 64 girls), participated in this study. These children have relocated from rural areas to urban centers such as Beijing, with a minimum residency period of 6 months. None of them reported a history of neurological or psychiatric disorders in the past 6 months. Among these participants, seven participants were excluded due to excessive head motion during image scanning, and 16 participants were excluded due to the lack of questionnaire information or questionnaire scores greater than ±3 standard deviation (SD). Finally, there were 101 participants aged from 10 to 14 were analyzed (47 boys and 54 girls, mean ± SD = 11.43 ± 0.91), and all of them were in the fifth or sixth grade, which correspond to the final 2 years of Chinese elementary school, similar to the last 2 years of elementary school and preceding junior high school in the international educational system; therefore, we mainly use preadolescent children to describe the participants in this study.

In addition, the control group which consists of 54 age- and socioeconomic status-matched children with nonmigrant background from two sites (Beijing and Chongqing) aged from 10 to 14 is included in our study to investigate whether there are any specific effects on the amygdala–prefrontal connectivity in preadolescent migrant children compared to the control.

This study was approved by the Institutional Review Board of the Faculty of Psychology at Beijing Normal University, with ethical approval number [BNU001912210084]. All participants and their caregivers provided informed consent prior to participation in the study.

### 2.2. Measurements

#### 2.2.1. Perceived Peer Victimization

Perceived peer victimization was quantified using a well-established Multidimensional Peer Victimization Scale [30] which assessed self-perceptions of being the target of peer aggression. It consists of four domains: verbal victimization, relational victimization, physical victimization, and property victimization. A composite perceived peer victimization score was calculated by the average across the four dimensions. A representative item is “Hurt me physically in some way.” The participants used a four-point scale ranging from 1 (never) to 4 (often) to describe their experiences. The perceived peer victimization scores greater than ±3 SD were excluded from analyses and higher scores indicated stronger feelings of being victimized by peers. The Cronbach's *α* coefficient in this study is 0.93. This scale has shown good validity and reliability among Chinese children and adolescents [31, 32].

#### 2.2.2. Self-Esteem

The Rosenberg Self-Esteem Scale [33] with a 10-item version (e.g., “On the whole, I am satisfied with myself”) was used to assess self-esteem. Participants rated their degree of agreement with the items using a four-point scale (1, strongly disagree; 2, disagree; 3, agree; 4, strongly agree). The self-esteem scores greater than ±3 SD were excluded and higher scores indicated stronger self-esteem. The Cronbach's *α* coefficient in this study is 0.83. The scale has demonstrated strong validity and reliability when used with Chinese children and adolescents [34].

#### 2.2.3. Depressive Symptoms

Depressive symptoms were assessed by the 20-item (e.g., “I felt down and unhappy”) short form of the Children's Depressive Symptoms Inventory (CES-DC) [35]. Each item was assessed with a four-point Likert scale rating from 1 to 4, where 1 signifies “not at all” and 4 indicates “a lot.” The depressive symptoms scores were calculated by the average of the 20 items and higher scores indicated more depressive feelings. The depressive symptoms scores greater than ±3 SD were excluded. The Cronbach's *α* coefficient in this study is 0.81. Chinese children and adolescents have consistently shown strong validity and reliability with this scale [36, 37].

### 2.3. Cortisol

Salivary cortisol samples were collected by Salivette sampling devices (Sarstedt, Germany) under the instruction of the experimenters, which followed our previous study [38]. To avoid contamination of saliva, the participants were asked not to change the oral environment including brushing their teeth, drinking, or eating at least 30 min before sampling. Each participant collected two saliva samples at 8:20 (i.e., morning cortisol) and 15:10 (i.e., afternoon cortisol), respectively, during three consecutive working days. None of the participants were taking hormone medications or had any diseases. After sampling, all cortisol samples were kept frozen (−25°C) until assay. Intra- and inter-assay variations of all of the cortisol samples were below 10%. The morning daily cortisol used in this study was computed by the average of cortisol collected at 8:20 AM over 3 days, which contains profiles of transition from the sleep state to the awake state and represents stable characteristics of the HPA-axis functioning. To ensure a normal distribution, cortisol values were log-transformed. In addition, the average morning cortisol values over 3 days greater than ±3 SD were defined outliers and excluded from further analyses.

### 2.4. Image Data Acquisition

Whole-brain resting-state images were acquired from Siemens 3.0T scanner (Magnetom Trio Trim syngo, Germany) using a 12-channel head coil with a T2*⁣*^*∗*^-sensitive echo-planar imaging (EPI) sequence based on blood oxygenation level-dependent (BOLD) contrast. In total, 33 axial slices (4.2 mm thickness, 0.7 mm skip) parallel to the anterior and posterior commissure (AC-PC) line and covering the whole brain were imaged with the following parameters: repetition time (TR) 2000 ms, echo time (TE) 30 ms, flip angle (FA) 90°; voxel size 3.1 × 3.1 × 4.2 mm [3], and field of view (FOV) 224 × 224 mm [2]. The resting-state scanning lasted 8 min and consisted of 240 volumes. During the resting-state scanning, participants were asked to keep their eyes open and remain still. High-resolution anatomical images were acquired through three-dimensional sagittal T1-weighted magnetization-prepared rapid gradient echo (MPRGE) with a total of 128 slices by the following parameters: TR, 2530 ms; TE, 3.39 ms; FA, 7°; inversion time (TI), 1100 ms; voxel size, 1 × 1 × 1.33 mm [3]; acquisition matrix, 256 × 256; FOV, 256 × 224 mm [2]; bandwidth (BW), 240Hz/Px; slice thickness, 1.33 mm.

### 2.5. Image Data Preprocessing

Brain images were preprocessed using statistical parametric mapping (SPM12) based on the MATLAB platform (version 8.1). The first and last five volumes of functional images were discarded due to participants' adaptation to scanning noise and signal equilibrium. The remaining images were corrected for slice acquisition timing and realigned for head motion correction. The participants whose maximum displacement (MD) was over 5 mm were excluded from further analyses, and all participants' frame-to-frame displacement (FD) was less than 0.5 mm. Subsequently, functional images were coregistered to each participant's gray matter image segmented from the corresponding highresolution T1-weighted image, then spatially normalized into a common stereotactic MNI space and resampled into 2-mm isotropic voxels. Finally, images were smoothed by an isotropic 3D-gaussian kernel with a 6-mm full-width half-maximum.

### 2.6. Amygdala Subregions

Given that the amygdala encompassed several subregions with different functional profiles, two major amygdala subregions, that is, the BLA and the CMA masks were created by using observer-dependent cytoarchitectonic probabilistic maps of the amygdala nuclei implemented in the Anatomy toolbox [39]. Maximum probability maps were used to create these anatomical masks using the Anatomy toolbox. Voxels were included in the maximum probability maps only if the probability of their assignment to either one amygdala subdivision was greater than 40% likelihood and higher than any other nearby structures. The BLA and CMA were as masks applied to the intrinsic seed-based functional connectivity analyses.

### 2.7. Intrinsic Seed-Based Functional Connectivity Analysis

Resting-state brain images were analyzed using seed-based whole-brain functional connectivity analysis with CMA and BLA as seeds, respectively. The regional time series of CMA or BLA were extracted from band-passed (0.008–0.10 Hz) images and then submitted to an individual fix-effect level general linear model analysis to access the intrinsic CMA or BLA seed-based functional connectivity with each of the other voxels in the whole brain. The Friston-24 head-motion parameters and the signals from white matter and cerebrospinal fluid (CSF) were included as nuisance covariates to account for physiological and movement-related artifacts. To evaluate whether perceived peer victimization was associated with intrinsic functional connectivity of the seed CMA/BLA, connectivity maps of the CMA/BLA were then submitted to a group-level multiple regression analysis correspondingly, with perceived peer victimization as the covariate of interest, and children's age, sex, as well as the mobility as the covariates of no interest. Mobility is a common indicator for preadolescent migrant children, which is calculated as an average score based on the frequency of their school transfers and relocations [40–42]. A higher score indicates greater mobility [43]. Significant clusters were determined by multiple comparison corrections at the cluster level with *p*  < 0.05 with Monte Carlo simulations using 3dClustSim module of AFNI [44]. According to this multiple correction method, the significant brain areas reported in our study meet the criterion that the initial uncorrected threshold of *p*  < 0.005 corresponding to 135 minimal cluster size or *p*  < 0.001 corresponding to 54 minimal voxels to reach the significance of cluster-level corrected *p*  < 0.05 based on a predefined automated anatomical labeling (AAL) frontal mask, which is the brain region we previously hypothesized to be functionally connected to the amygdala subregions [45]. Mean parameter estimates that represent connectivity strength were extracted from significant clusters to examine the relationship between perceived peer victimization and CMA/BLA functional connectivity. In order to conduct additional analyses to examine whether there were convergent findings, we also used the same method to extract values from the prefrontal mask, that is, superior frontal gyrus and middle frontal gyrus from the unbiased AAL template in preadolescent migrant children and control group children.

### 2.8. Mediation and Moderation Analysis

The classic mediation model and the moderated mediation model were applied to test our hypotheses using the PROCESS macro in SPSS [46]. To investigate whether children's perceived peer victimization (M, the mediator variable) mediated the relation between CMA/BLA seed-based functional connectivity (X, the independent variable) and depressive symptoms (Y, the dependent variable), we conducted a classic mediation model. Furthermore, whether there was a moderating effect of self-esteem or cortisol based on top of the mediation effect of perceived peer victimization was evaluated using moderated mediation model, which adds the moderator variable (i.e., W) based on the classic mediation model and has been successfully employed in previous studies [47]. All the models above were tested with a 5000 bias-corrected bootstrapping resampling approach, which generated a 95% confidence interval (CI). Besides, children's age, gender, and mobility were covariates in all models above.

## 3. Results

### 3.1. Descriptive Statistics and Correlation of All Variables

The mean values, SDs of each measurement variable, and the correlation coefficients between variables are shown in Table 1. As seen, perceived peer victimization is positively correlated with BLA/CMA-SFG/dlPFC connectivity and depressive symptoms. Self-esteem is negatively correlated with depressive symptoms. Morning daily cortisol is negatively correlated with peer victimization.

### 3.2. Higher Perceived Peer Victimization Linked to Amygdala–Prefrontal Hyperconnectivity

**Figure 1:**
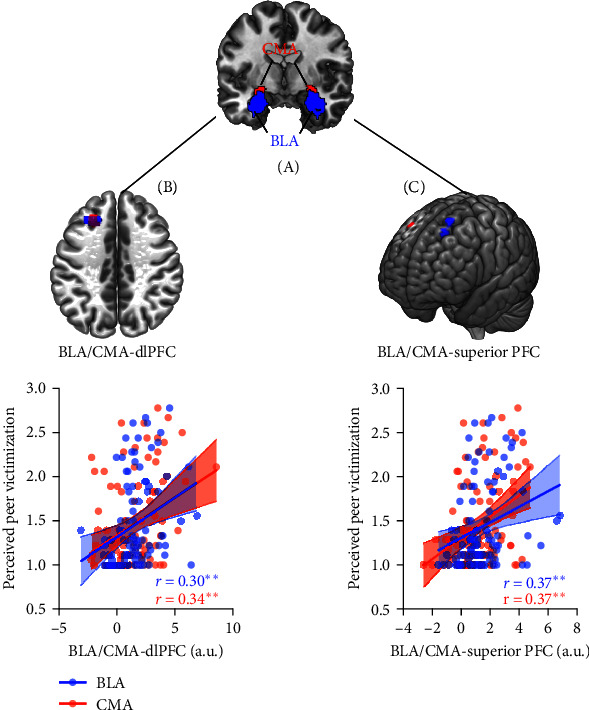
Children with higher intrinsic connectivity of both centromedial amygdala (CMA) and basolateral amygdala (BLA) with the dorsolateral PFC (dlPFC) and superior PFC perceived higher levels of peer victimization. (A) A representative coronal slice shows the CMA and BLA. The left CMA (red) and BLA (blue) inside the circled area were used as seeds for connectivity analyses. (B) Significant clusters in the dlPFC showed positive correlations between intrinsic CMA/BLA-dlPFC connectivity and perceived peer victimization, as well as (C) between intrinsic CMA/BLA-superior PFC connectivity and perceived peer victimization when regressing out age, sex, and mobility. *⁣*^*∗∗*^*p* < 0.01; a.u., arbitrary unites.

To examine how the perceived peer victimization alters the intrinsic functional connectivity of the BLA and CMA, we conducted multiple regression analyses for BLA and CMA seed-based (Figure 1A) functional connectivity maps, with perceived peer victimization as the covariate of interest, and children's age, sex, and mobility as the covariates of no interest. This analysis revealed significant clusters in the dorsolateral prefrontal cortex (dlPFC) extending into the superior prefrontal gyrus (SFG), showing higher connectivity with the BLA in children with higher perceived peer victimization (Figure 1B,C, and Supporting Information 1: Table S1). For the CMA-based functional connectivity, we found a similar neural pattern (Figure 1B,C, and Supporting Information 1: Table S1). In short, higher perceived peer victimization is associated with greater BLA/CMA connectivity with dlPFC and SFG in preadolescent migrant children.

**Figure 2:**
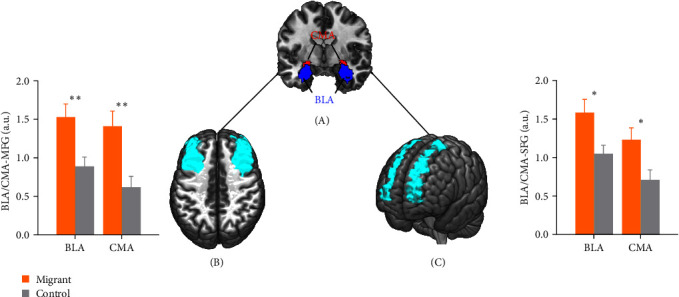
Compared preadolescent migrant children with children from a nonmigrant background in the intrinsic connectivity of both centromedial amygdala (CMA) and basolateral amygdala (BLA) with the dorsolateral PFC (MFG/dlPFC) and superior PFC(SFG). (A) A representative coronal slice shows the CMA and BLA. The left CMA (red) and BLA (blue) inside the circled area were used as seeds for connectivity analyses. (B) Intrinsic CMA/BLA-MFG connectivity. (C) Intrinsic CMA/BLA-SFG connectivity. *⁣*^*∗∗*^*p*  < 0.01; *⁣*^*∗*^*p*  < 0.05.

Further, to verify whether there are specific effects on the amygdala-PFC hyperconnectivity, we conducted an independent samples *t*-test to examine differences in amygdala connectivity patterns between the migrant group and control group. Utilizing the control group allows us to investigate the differences in CMA/BLA connectivity patterns more consistently while accounting for potential regional differences that may affect our findings while also considering regional influences [48]. We extracted values from two prefrontal masks, that is, superior frontal gyrus and middle frontal gyrus from the unbiased AAL template in preadolescent migrant children and control group. As expected, this analysis revealed that migrant children showed significantly stronger connectivity between CMA/BLA and prefrontal regions including middle PFC and superior PFC as compared to age-matched controls (Figure 2). Preadolescent migrant children might have a higher CMA/BLA-functional connectivity with middle PFC and superior PFC, which in turn leads to a greater perception of peer victimization. In addition, similar patterns were observed among migrant children and children without migration background when analyzing data from two separate sites (Beijing: *n* = 28; Chongqing: *n* = 26). Notably, there were no significant differences between the amygdala–prefrontal hyperconnectivity of children without migration background in Beijing and Chongqing site (*P*_*S*_ > 0.05) (details can be seen in Supporting Information). Together, these results indicate preadolescent migrant children showed significantly stronger connectivity between CMA/BLA and prefrontal regions as compared to age-matched children from a nonmigrant background

### 3.3. Competition Model About Amygdala–Prefrontal Hyperconnectivity, Perceived Peer Victimization, and Depressive Symptoms

**Figure 3:**
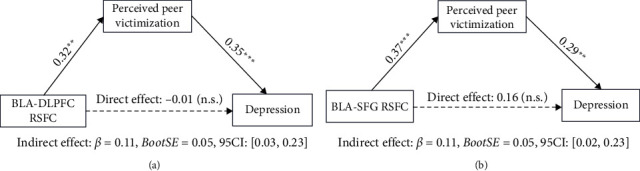
BLA seed-based functional connectivity (X) affects depressive symptoms (Y) through perceived peer victimization (M). (A) A mediation model demonstrated a mediatory role of perceived peer victimization on the association between intrinsic BLA-DLPFC connectivity and depressive symptoms. (B) A mediation model showed that BLA-SFG connectivity affected depressive symptoms through perceived peer victimization. Paths are marked with standardized coefficients. The whole model controls three covariables: age, sex, and mobility. Notes: RSFC, resting-state functional connectivity; BLA, basolateral amygdala; dlPFC, dorsolateral PFC; SFG, superior PFC; *⁣*^*∗∗*^*p*  < 0.01; *⁣*^*∗∗∗*^*p*  < 0.001.

We then examined whether BLA/CMA-dlPFC/SFG connectivity affects children's depressive symptoms via perceived peer victimization by implementing mediation analyses. This analysis revealed that both increased BLA-dlPFC functional connectivity (*β* = 0.11, SE = 0.05, 95% CI = [0.03, 0.23], Figure 3A) and BLA-SFG connectivity (*β* = 0.11, SE = 0.05, 95% CI = [0.02, 0.23], Figure 3B) affected children's higher depressive symptoms through perceived higher peer victimization. Parallel mediation analysis for CMA-prefrontal connectivity revealed a similar effect, that is, both increased CMA-dlPFC functional connectivity (*β* = 0.11, SE = 0.05, 95% CI = [0.03, 0.22], Supporting Information 2: Figure S1) and CMA-SFG connectivity (*β* = 0.14, SE = 0.06, 95% CI = [0.03, 0.26], Supporting Information 2: Figure S1) could also account for children's higher depressive symptoms through perceived higher peer victimization. Moreover, we also observed a chain mediation effect that the BLA-SFG connectivity affected the CMA-SFG connectivity first, then related to the depressive symptoms through perceived peer victimization (Supporting Information 3: Figure S2). These results indicate that children's perceived peer victimization mediates the relation between CMA/BLA intrinsic functional connectivity with dlPFC/SFG and depressive symptoms in preadolescent migrant children.

Considering some research suggests that adversity can affect the brain and cause internalization problems [49], we also tested the competition model that whether peer victimization could affect amygdala–prefrontal hyperconnectivity and lead to depressive symptoms. Thus, we examined whether amygdala–prefrontal hyperconnectivity mediated the association between perceived peer victimization and depressive symptoms. The mediation analysis revealed that the relationship between perceived peer victimization and depressive symptoms could not be mediated by the BLA-dlPFC functional connectivity (*β* = 0.01, SE = 0.03, 95%CI = [−0.07,0.06]) and BLA-SFG connectivity (*β* = 0.05, SE = 0.04, 95% CI = [−0.03,0.14]). Similarly, perceived higher peer victimization could not also account for children's higher depressive symptoms through both increased CMA-dlPFC functional connectivity (*β* = 0.01, SE = 0.04, 95%CI = [−0.07, 0.09]) and CMA-SFG connectivity (*β* = −0.06. SE = 0.04, 95%CI = [−0.15.0.02]). Together, these results indicate that children's perceived peer victimization mediates the relation between CMA/BLA intrinsic functional connectivity with dlPFC/SFG and depressive symptoms in preadolescent migrant children.

### 3.4. Self-Esteem and Morning Daily Cortisol Moderated the Mediation Effect of Perceived Peer Victimization

**Figure 4:**
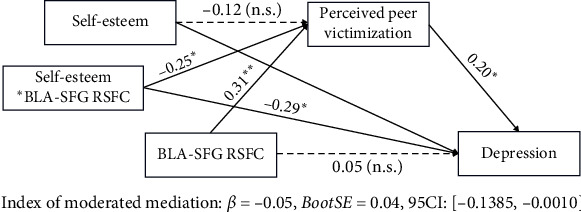
A schematic illustration of moderated mediation analyses with self-esteem as moderator. Paths are marked with standardized coefficients. The whole model controls three covariables: age, sex, and mobility. RSFC, resting-state functional connectivity; BLA, basolateral amygdala; SFG, superior PFC; *⁣*^*∗*^*p*  < 0.05; *⁣*^*∗∗*^*p*  < 0.01.

To investigate whether self-esteem moderates the mediation effect of perceived peer victimization on the relationship between BLA/CMA-prefrontal connectivity and depressive symptoms, we applied moderated mediation models to examine the buffering effect of self-esteem. We found that self-esteem moderated the relation between higher BLA-SFG connectivity and higher perceived peer victimization, which in turn affected higher depressive symptoms in preadolescent migrant children (*β* = −0.05, SE = 0.04, 95%CI = [−0.139, −0.001], Figure 4). The mediation effect of perceived peer victimization on the relation between BLA-SFG connectivity and depressive symptoms only exists in preadolescent migrant children with low self-esteem.

**Figure 5:**
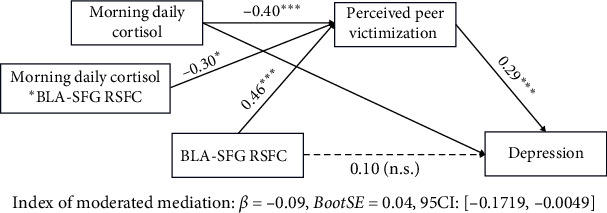
A schematic illustration of moderated mediation analyses with morning daily cortisol as moderator. Paths are marked with standardized coefficients. The whole model controls three covariables: age, sex, and mobility. RSFC, resting-state functional connectivity; BLA, basolateral amygdala; SFG, superior PFC; *⁣*^*∗*^*p*  < 0.05; *⁣*^*∗∗∗*^*p*  < 0.001.

To further investigate whether basal cortisol moderated the mediation effect of perceived peer victimization on the relation between BLA/CMA-prefrontal connectivity and depressive symptoms, we also examined the buffering effect of morning basal cortisol. We found that basal cortisol level moderated the relation between higher BLA-SFG connectivity and higher perceived peer victimization, which in turn affected higher depressive symptoms in preadolescent migrant children (*β* = −0.08, SE = 0.04, 95%CI = [−0.158, −0.008], Figure 5). The mediation effect of perceived peer victimization on the relation between BLA-SFG connectivity and depressive symptoms only exists in preadolescent migrant children with low morning daily cortisol. Additionally, we controlled for the potential effects of the menstrual period on cortisol levels by including a covariate for menstrual status (0 for none, 1 for menstruating; four participants reported being in their menstrual period), and the significance of the cortisol-related moderated mediation analysis remained robust.

These results indicate that the mediation effect of perceived peer victimization on the relation between BLA-SFG connectivity and depressive symptoms only holds in preadolescent migrant children with low self-esteem or low morning daily cortisol.

## 4. Discussion

In this study, we investigate how the amygdala intrinsic functional connectivity links to perceived peer victimization and depressive symptoms in preadolescent migrant children. We found that children with greater amygdala intrinsic connectivity with SFG and dlPFC exhibited higher perceived peer victimization, which mediated higher depressive symptoms in preadolescent migrant children. Critically, higher BLA connectivity with the SFG acted as potentially vulnerable factors linking higher peer victimization and greater risk for depressive symptoms in children with low self-esteem or low daily cortisol. Our findings suggest that the amygdala–SFG intrinsic connectivity acts as a sensitive neural circuitry and its vulnerability could be buffered by the protective effects of self-esteem and cortisol in preadolescent migrant children with early adversity.

With neuroimaging data, we found higher peer victimization jointly corresponded with greater amygdala intrinsic connectivity with the superior PFC and dlPFC that are critical for emotion regulation [50]. Increased resting-state connectivity of the amygdala with dlPFC was found to be positively correlated with perceived aggression [51], which is in line with the concept of peer victimization. Previous studies have also revealed that greater amygdala–SFG connectivity was associated with lower positive affect and higher depressive symptoms [52]. These results implicate that the obstacle of emotional regulation circuits might be the antecedent of perceiving peer victimization. Our findings provide preliminary evidence that stronger amygdala connectivity may serve as a potential neural vulnerable factor in perceiving peer victimization among Chinese preadolescent migrant children. Previous studies have suggested that perceived peer victimization is associated with impaired corticolimbic functioning during the processing of negative emotional information [53, 54]. These results are consistent with previous studies that connections between the amygdala and the PFC are sensitive to a wide range of adversity [55]. Our findings also support the notion that a more mature connectivity profile relates to perceived stress [56]. Findings are also in keeping with social–emotional models of development which proposed that a lack of emotional competence may leave youth vulnerable to poor social interactions and negative peer relationships, such as those characterized by perceived peer victimization [57]. Second, the mediation results support that perceived stress mediates the impact of brain activity on depressive symptoms [58], rather than stress impacts the brain activity that leads to depressive symptoms [49]. This finding suggests that alterations in the functioning of the fronto-limbic circuitry during emotion regulation might be a vulnerability marker for sensitivity to the development of perceived peer victimization and depressive symptoms among preadolescent migrant children. This adds weight to the stress susceptibility model [59].

Furthermore, we found that the amygdala connectivity of preadolescent migrant children is higher than that of nonmigrant children, which may indicate that children who migrate from rural to urban areas may become more attuned to peer relationships as a result of living in socially disadvantaged environments. As a result, they may be more susceptible to experiencing peer victimization, which were consistent with the previous study [3].

More importantly, our results demonstrate that higher self-esteem and morning daily cortisol could buffer the link of amygdala–prefrontal hyperconnectivity to perceived peer victimization and depressive symptoms. These results are in line with previous studies investigating self-esteem might buffer the brain mechanisms of perceived stress (i.e., poverty) [23] and modulate the neural responses to social cues [60]. In addition, this study revealed that basal cortisol at certain time window(s) could buffer the amygdala connectivity of perceived stress to our knowledge, suggesting a protective role of cortisol [61]. This finding aligned with previous research that cortisol might be related to amygdala-centered functional connectivity and perceived anxiety [62]. One may assume that individual positive adaptation is dependent on the engagement of the slow response of the HPA-axis system, while cortisol, as the end-product of the HPA axis, is greatly sensitive to stress and could provide energy support for potential environmental challenges [63]. Such a pattern of cortisol and the amygdala connectivity could be seen in a population exposed to other early life stress [64].

Interestingly, we observed that the moderating effect of self-esteem and morning daily cortisol is on the pathway of BLA-SFG connectivity, but not on the CMA-SFG functional coupling. Different portions of the amygdala have distinct functions with BLA as a gateway of the amygdala to receiving sensory inputs while CMA is the major output of the amygdala to express emotional and physiological responses [65]. Peer victimization could be perceived as one kind of social threat and strong stress perception, especially for preadolescent migrant children [66]. This result may inspire us that these two protective factors could merely have buffering roles when perceiving social threats rather than generating responses. Recently, animal studies have converged on the evidence that BLA has wide projection with sensory and prefrontal regions including the SFG to regulate the stress effects on brain anatomy and function [67]. Besides, as a key subregion of the amygdala, BLA may communicate with the hippocampus and the PFC to modulate the long-term memory consolidation of life events by regulating and integrating the norepinephrine and glucocorticoid levels [68], which may contribute to the emotional arousal of daily life events and peer victimization perceiving for preadolescent migrant children. Indeed, the BLA-mPFC circuitry is considered to be one basis of depressive-like behaviors [69, 70] and the activation of the BLA-mPFC neurons also has a prodepressive impact [71]. The higher BLA-SFG functional connectivity is possibly involved in perceiving peer victimization and maintaining threat-related information in preadolescent migrant children, and finally induces internalizing depressive symptoms tendency, while higher self-esteem and higher morning cortisol could provide both mental resources and endocrinal resources to ameliorate these negative effects, which may account for the above-moderating effects existing only in BLA-SFG connectivity.

Together, our data converge onto a neurobiological model that amygdala–prefrontal hyperconnectivity could affect higher depressive symptoms through perceived peer victimization in preadolescent migrant children. Critically, higher self-esteem and daily cortisol may buffer the link of amygdala-SFG intrinsic circuitry to perceived peer victimization and depressive symptoms.

Our study has several limitations. First, we focused on amygdala-centric intrinsic functional networks and cannot rule out the possibility that significant findings may be observed in other networks. Second, we focused our study on a sample of Chinese preadolescent migrant children. While this may initially appear to limit our ability to detect contrast differences with other children, we argue that it is actually a strength of our research. By focusing on this understudied population, we are able to provide nuanced insights into their unique experiences and challenges. However, we acknowledge that our findings may not be fully generalizable to all children, and we encourage future research that explores similar phenomena across different populations and contexts. Third, the mediation analysis presented in this study has certain limitations due to the lack of a follow-up study to establish chronicity. Therefore, further longitudinal studies are needed to reveal causal relationships.

Our study demonstrates that amygdala–prefrontal hyperconnectivity may serve as a potentially vulnerable factor sensitive to perceived peer victimization and depressive symptoms during preadolescence and highlights the protective effects of self-esteem and cortisol on stress perception and depressive symptoms. By understanding the vulnerable neurobiological indicators associated with the perception of adversity and its impact on psychological development, as well as two important physiological and psychological buffering agents, we can design targeted interventions that address the unique needs of children from disadvantaged backgrounds facing these challenges.

## Figures and Tables

**Table 1:** The mean values, standard deviations of each measurement variable, and the correlation coefficients between variables.

Variable	1	2	3	4	5	6	7	8	9	10	11
1. Age	—	—	—	—	—	—	—	—	—	—	—
2. Gender	0.07	—	—	—	—	—	—	—	—	—	—
3. Mobility	0.05	−0.20*⁣*^*∗*^	—	—	—	—	—	—	—	—	—
4. BLA-dlPFC connectivity	−0.21*⁣*^*∗*^	−0.00	−0.08	—	—	—	—	—	—	—	—
5. BLA-SFG connectivity	−0.21*⁣*^*∗*^	−0.09	−0.06	0.75*⁣*^*∗∗∗*^	—	—	—	—	—	—	—
6. CMA-dlPFC connectivity	−0.18	−0.04	0.03	0.70*⁣*^*∗∗∗*^	0.56*⁣*^*∗∗∗*^	—	—	—	—	—	—
7. CMA-SFG connectivity	−0.15	−0.08	0.03	0.57*⁣*^*∗∗∗*^	0.51*⁣*^*∗∗∗*^	0.74*⁣*^*∗∗∗*^	—	—	—	—	—
8. Peer victimization	−0.04	−0.24	0.08	0.30*⁣*^*∗∗*^	0.37*⁣*^*∗∗∗*^	0.34*⁣*^*∗∗∗*^	0.37*⁣*^*∗∗∗*^	—	—	—	—
9. Depressive symptoms	0.16	0.06	−0.10	0.07	0.22*⁣*^*∗*^	0.13	−0.02	0.31*⁣*^*∗∗*^	—	—	—
10. Self-esteem	−0.24*⁣*^*∗*^	0.10	0.02	−0.07	−0.19	−0.07	0.09	−0.17	−0.49*⁣*^*∗∗∗*^	—	—
11. Morning daily cortisol	0.25*⁣*^*∗*^	0.09	−0.10	0.09	0.01	−0.11	−0.12	−0.32*⁣*^*∗∗*^	−0.07	−0.01	—
*M*	11.43	—	0.91	1.53	1.58	1.41	1.23	1.45	0.68	3.11	0.23
SD	0.91	—	0.83	1.71	1.75	1.99	1.55	0.50	0.37	0.49	0.33

*⁣* ^*∗*^ *p* < 0.05, *⁣*^*∗∗*^*p* < 0.01, and *⁣*^*∗∗∗*^*p* < 0.001.

## Data Availability

The data that support the findings of the study are available from the corresponding author (Danhua Lin) upon reasonable request.

## Supporting Information

Supporting Information 1 Table S1: presenting all target regions of CMA and BLA intrinsic connectivity significantly correlating with children's perceived peer victimization. Supporting Information Results section providing the significant differences in amygdala connectivity patterns between preadolescent migrant children and 54 children without migration background from two sites (Beijing and Chongqing) aged from 10–14. A Supporting Information References section supporting the Supporting Information Table section.

Supporting Information 2 Figure S1: presenting the mediation model that CMA seed-based functional connectivity could affect depressive symptoms through perceived peer victimization.

Supporting Information 3 Figure S2: presenting the chain mediation model that BLA-SFG RSFC could affect depressive symptoms through intrinsic CMA-SFG connectivity and perceived peer victimization.
