# Supplementary material for: Psychophysiological Factors Moderate Amygdala–Prefrontal Connectivity Linked to Perceived Peer Victimization and Depressive Symptoms in Preadolescent Migrant Children
Source: Depress Anxiety. 2024 Oct 10;2024:5596651. doi: 10.1155/2024/5596651 (PMC11919204; doi:10.1155/2024/5596651)
Supplement: Supporting Information 1 — Table S1: presenting all target regions of CMA and BLA intrinsic connectivity significantly correlating with children's perceived peer victimization. Supporting Information Results section providing the significant differences in amygdala connectivity patterns between preadolescent migrant children and 54 children without migration background from two sites (Beijing and Chongqing) aged from 10–14. A Supporting Information References section supporting the Supporting Information Table section. [file 5596651.f1.docx]

**Supplementary Materials**

**Supplementary Table 1**

**Table S1. Target regions of CMA and BLA intrinsic connectivity significantly correlated with children’s perceived peer victimization.**

| **Seed** | **Target Regions** | **L/R** | **BA** | **Voxel** | ***T* value** | **MNI (x y z)** |
| --- | --- | --- | --- | --- | --- | --- |
| CMA | Putamen | L | 34 | 242 | 4.25 | -20 2 -10 |
|  | Middle Temporal Gyrus | R | 13 | 252 | 3.80 | 44 -46 18 |
|  | Cingulate Gyrus | R | 23 | 346 | 4.12 | 14 -36 36 |
|  | Superior Medial Frontal | L | 32 | 198 | 4.07^a^ | -10 26 36 |
|  | Superior Frontal Gyrus | R | 8 | 181 | 3.55^a^ | 26 26 52 |
|  | Precuneus | L | 7 | 194 | 3.99 | -10 -54 50 |
| BLA | Putamen | L | 13 | 375 | 4.18 | -24 6 -10 |
|  | Cuneus | L | 18 | 151 | 3.72^a^ | -2 -84 22 |
|  | Middle Frontal Gyrus | L | 9 | 121 | 3.54^a^ | -24 30 38 |
|  | Middle Cingulum Gyrus | R | 31 | 150 | 3.80 | 12 -26 32 |
|  | Precentral | L | 6 | 240 | 4.71 | -38 -8 48 |
|  | Superior Frontal Gyrus | L | 6/8 | 162 | 3.51^a^ | -6 12 50 |

^a^ Given our prior hypothesis, significant clusters in the prefrontal cortex were determined by multiple comparison corrections at the cluster level with *p* < 0.05 using 3dClustSim with an anatomically defined prefrontal mask from AAL template. CMA, centromedial amygdala; BLA, basolateral amygdala; BA, Brodmann’s area; MNI, Montreal Neurological Institute.

Notably, communication of putamen with the amygdala processes represent anxiety-specific markers rather than depression markers^1,2,3^. Thus, we only focus on the amygdala-prefrontal functional connectivity in our study.

**Supplementary Results**

**3.1 Higher perceived peer victimization linked to amygdala-prefrontal hyperconnectivity**

Further, to verify whether there are specific effects on the amygdala-PFC hyperconnectivity, we conducted an independent samples t-test to examine differences in amygdala connectivity patterns between preadolescent migrant children and 54 children without migration background from two sites (Beijing and Chongqing) aged from 10-14. Specifically, the results indicated a significant difference in BLA-dlPFC functional connectivity between control group (M=1.53, SD=1.71) and preadolescent migrant children group (M=0.89 SD=0.87), *t*(153)=-3.10, *p* =0.002, a significant difference in BLA-SFG functional connectivity between control group (M=1.05, SD=0.83) and preadolescent migrant children group (M=1.58, SD=1.75), *t*(151)=-2.59, *p*=0.011, a significant difference in CMA-dlPFC functional connectivity between control group (M=0.62, SD=1.00) and preadolescent migrant children group (M=1.41, SD=1.99), *t*(153)=-3.28, *p*=0.001, a significant difference in CMA-SFG functional connectivity between control group (M=0.71, SD=0.95) and preadolescent migrant children group (M=1.23, SD=1.54), *t*(150)=-2.58, *p*=0.011.

We also found significant differences in amygdala-prefrontal functional connectivity between preadolescent migrant children group and control group in Chongqing site or Beijing site.

Specifically, we found a significant difference in BLA-dlPFC functional connectivity between control group in Chongqing site (M=0.95, SD=0.82) and preadolescent migrant children group (M=1.53, SD=1.71), *t*(85)=-2.51, *p*=0.014; a marginal significance in BLA-SFG functional connectivity between control group in Chongqing site (M=112, SD=0.91) and preadolescent migrant children group (M=1.58, SD=1.75), *t*(78)=-1.88, *p*=0.064; a significant difference in CMA-dlPFC functional connectivity between control group in Chongqing site (M=0.56, SD=0.85) and preadolescent migrant children group (M=1.41, SD=1.99), *t*(97)=3.26, *p* =0.002; a significant difference in CMA-SFG functional connectivity between control group in Chongqing site (M=0.57, SD=0.80) and preadolescent migrant children group（M=1.23, SD=1.54), *t*(78)= -3.03, *p*=0.003.

We found a significant difference in BLA-dlPFC functional connectivity between control group in Beijing site (M=0.84, SD=0.93) and preadolescent migrant children group (M=1.53, SD=1.71), *t*(82)=-2.84, *p*=0.006; a significant significance in BLA-SFG functional connectivity between control group in Beijing site (M=0.98, SD=0.76) and preadolescent migrant children group (M=1.58, SD=1.75), *t*(105)=-2.67, *p*=0.009; a significant difference in CMA-dlPFC functional connectivity between control group in Beijing site (M=0.68, SD=1.13) and preadolescent migrant children group (M=1.41, SD=1.99), *t*(78)=-2.52, *p*=0.014; a non-significant difference in CMA-SFG functional connectivity between control group in Beijing site (M=0.85, SD=1.07) and preadolescent migrant children group (M=1.23, SD=1.54), *t*(62)=-1.51, *p*=0.138.

**Supplementary Figures**

**
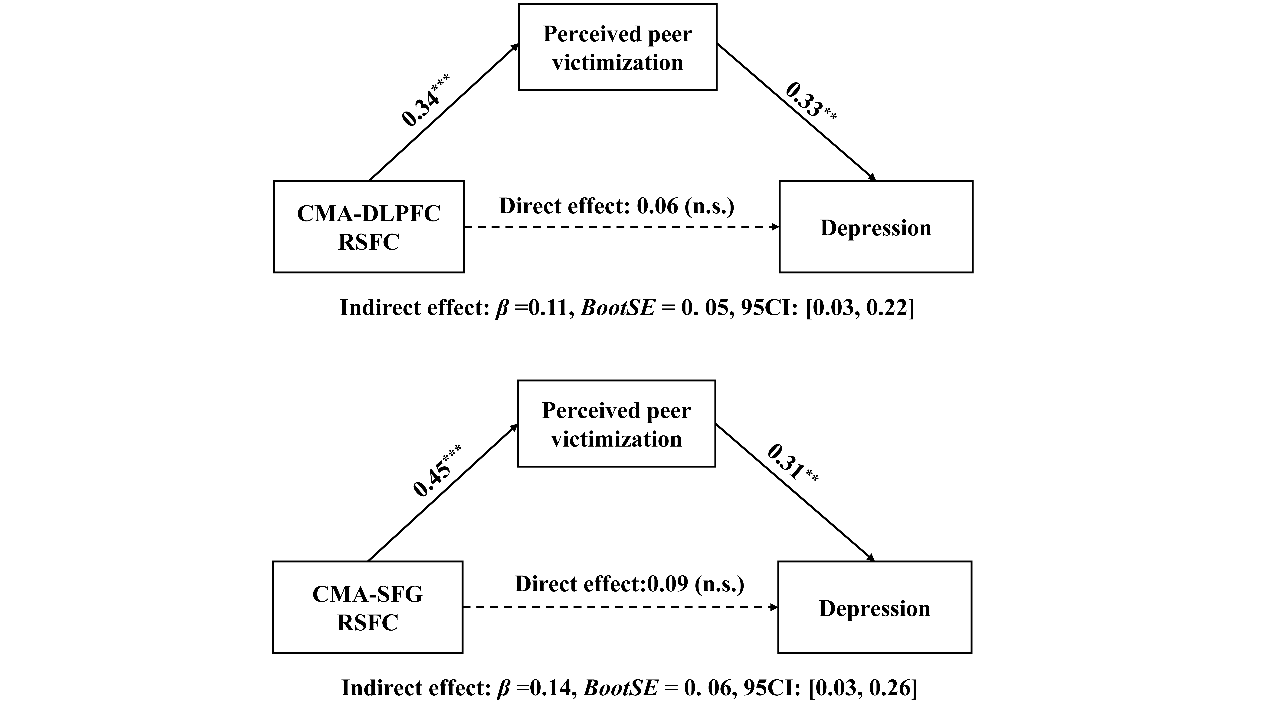
**

**Figure S1. CMA seed-based functional connectivity (X) affecting depression (Y) through perceived peer victimization (M).** (A) A mediation model showed demonstrated a mediatory role of perceived peer victimization on the association between intrinsic CMA-DLPFC connectivity and depression. (B) A mediation model showed that CMA-SFG connectivity affected depression though perceived peer victimization. Paths are marked with standardized coefficients. The whole model controls three covariables: age, sex and mobility. Notes: RSFC, resting-state functional connectivity; CMA, centromedial amygdala; DLPFC, dorsolateral PFC; SFG, superior PFC; **p < 0.05*; ***p < 0.01; ***p < 0.001.*

**
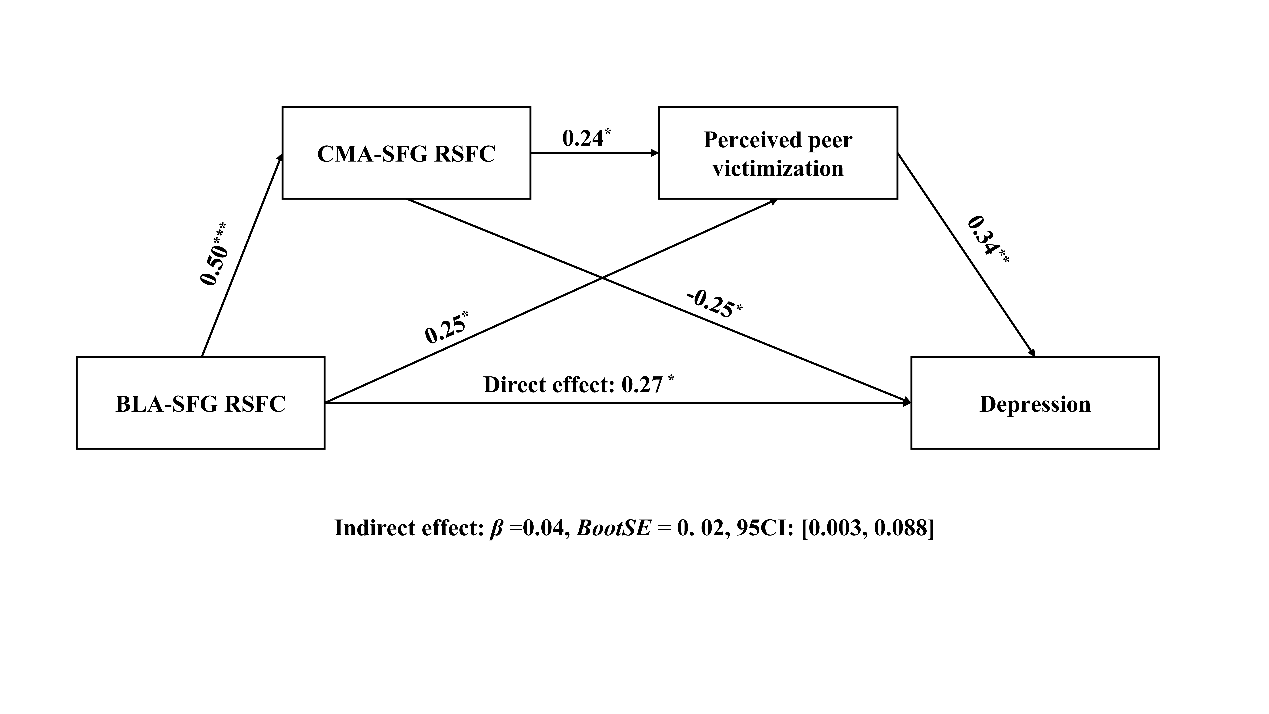
Figure S2. BLA-SFG RSFC (X) affecting depression (Y) through intrinsic CMA-SFG connectivity (M1) and perceived peer victimization (M2).** Paths are marked with standardized coefficients. The whole model controls three covariables: age, sex and mobility. Notes: RSFC, resting-state functional connectivity; BLA, basolateral amygdala; CMA, centromedial amygdala; SFG, superior PFC; **p < 0.05*; ***p < 0.01; ***p < 0.001.*

**References**

1. Qin S, Young CB, Duan X, Chen T, Supekar K, Menon V. Amygdala subregional structure and intrinsic functional connectivity predicts individual differences in anxiety during early childhood. *Biological psychiatry* 2014; 75(11): 892-900.

2. Wang, C., Wang, Y., Lau, W. K., Wei, X., Feng, X., Zhang, C., ... & Zhang, R. Anomalous static and dynamic functional connectivity of amygdala subregions in individuals with high trait anxiety. *Depression and Anxiety* 2021; *38*(8): 860-873.

3. Xu, X., Dai, J., Chen, Y., Liu, C., Xin, F., Zhou, X., ... & Becker, B. Intrinsic connectivity of the prefrontal cortex and striato-limbic system respectively differentiate major depressive from generalized anxiety disorder. *Neuropsychopharmacology* 2021; *46*(4): 791-798.
