# Supplementary figures and images for: Psychophysiological Factors Moderate Amygdala–Prefrontal Connectivity Linked to Perceived Peer Victimization and Depressive Symptoms in Preadolescent Migrant Children
Source: Depress Anxiety. 2024 Oct 10;2024:5596651. doi: 10.1155/2024/5596651 (PMC11919204; doi:10.1155/2024/5596651)

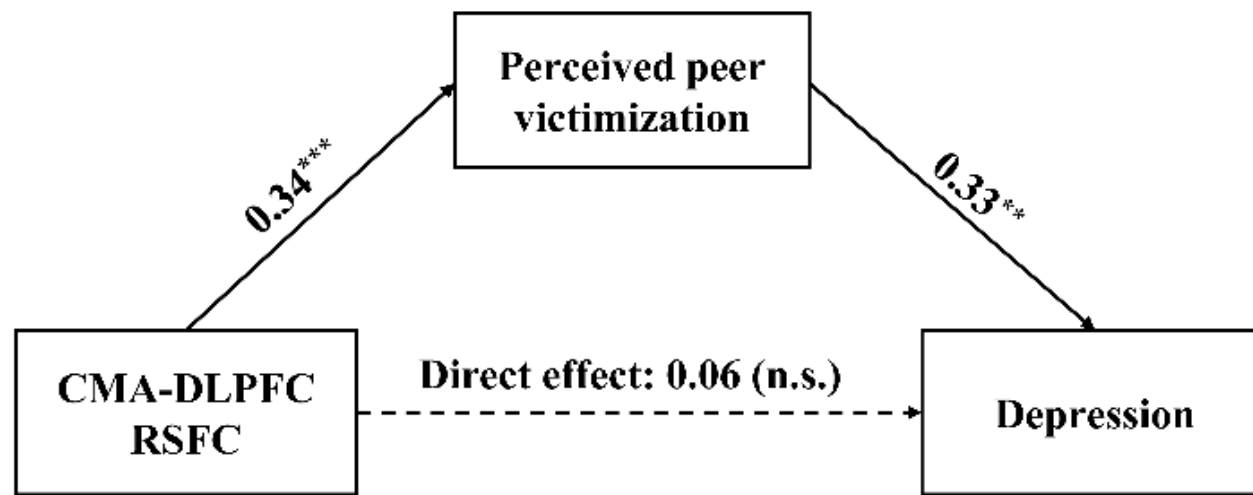

Indirect effect:  $\beta = 0.11$ ,  $BootSE = 0.05$ , 95CI: [0.03, 0.22]

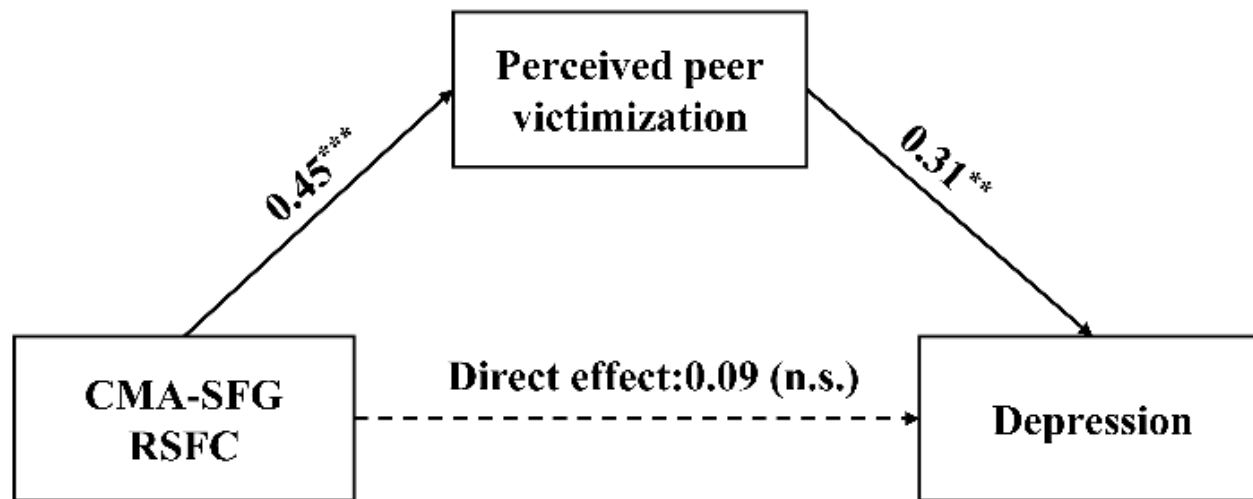

Indirect effect:  $\beta = 0.14$ ,  $BootSE = 0.06$ , 95CI: [0.03, 0.26]

Supplement: Supporting Information 2 — Figure S1: presenting the mediation model that CMA seed-based functional connectivity could affect depressive symptoms through perceived peer victimization. [file 5596651.f2.pdf]

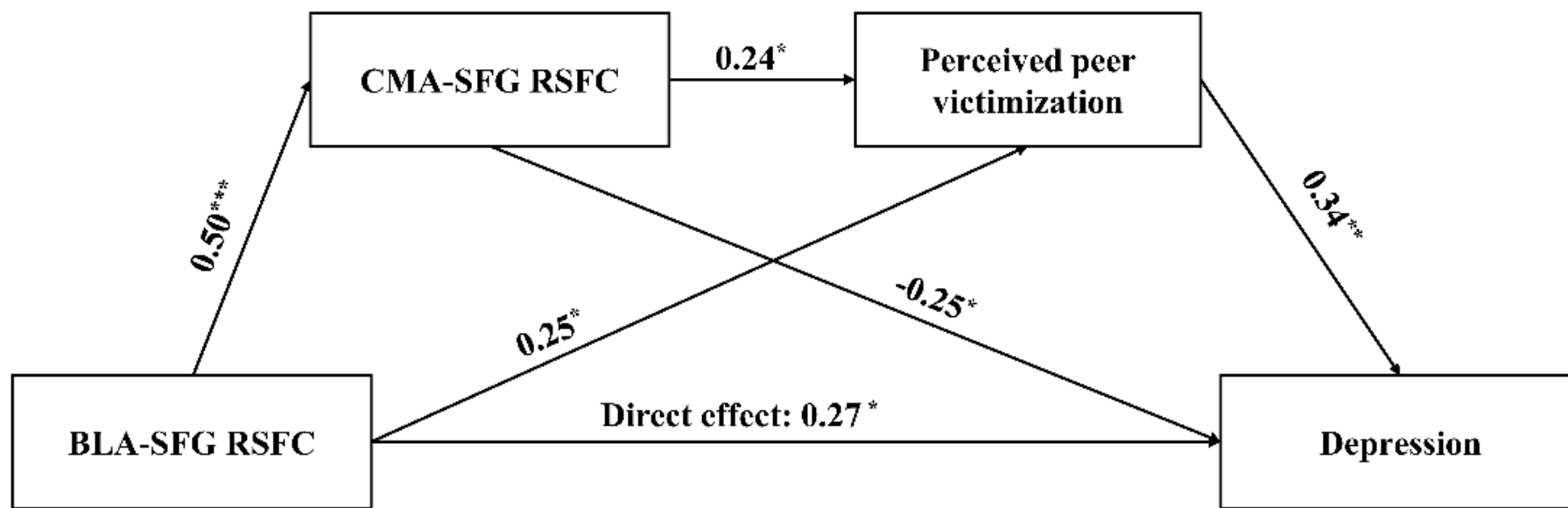

Indirect effect:  $\beta = 0.04$ ,  $BootSE = 0.02$ , 95CI: [0.003, 0.088]

Supplement: Supporting Information 3 — Figure S2: presenting the chain mediation model that BLA-SFG RSFC could affect depressive symptoms through intrinsic CMA-SFG connectivity and perceived peer victimization. [file 5596651.f3.pdf]
